# The Performance of HE4 Alone and in Combination with CA125 for the Detection of Ovarian Cancer in an Enriched Primary Care Population

**DOI:** 10.3390/cancers14092124

**Published:** 2022-04-24

**Authors:** Chloe E. Barr, Garth Funston, David Jeevan, Sudha Sundar, Luke T. A. Mounce, Emma J. Crosbie

**Affiliations:** 1 Manchester Academic Health Science Centre, Department of Obstetrics and Gynaecology, St Mary’s Hospital, Manchester University NHS Foundation Trust, Manchester M13 9WL, UK; chloe.barr@mft.nhs.uk; 2 The Primary Care Unit, Department of Public Health and Primary Care, University of Cambridge, Cambridge CB1 8RN, UK; gf272@medschl.cam.ac.uk; 3 Centre for Primary Care and Health Service Research, Faculty of Biology, Medicine and Health, University of Manchester, Manchester M13 9WL, UK; 4 Institute of Cancer and Genomic Sciences, College of Medical and Dental Science, University of Birmingham, Birmingham B15 2TT, UK; david.jeevan@nhs.net (D.J.); s.s.sundar@bham.ac.uk (S.S.); 5 Institute of Health Research, College of Medicine and Health, University of Exeter Medical School, Exeter EX1 2LU, UK; l.t.a.mounce@exeter.ac.uk; 6 Division of Cancer Sciences, Faculty of Biology, Medicine and Health, University of Manchester, Manchester M13 9WL, UK

**Keywords:** ovarian cancer, early detection, primary care, biomarker, HE4, CA125, ROMA

## Abstract

**Simple Summary:**

Ovarian cancer is the most common cause of death from gynaecological cancer in the UK. Survival is better when the disease is diagnosed early. However, identifying ovarian cancer is challenging because symptoms are non-specific. Simple, accurate tests are needed to help identify ovarian cancer in symptomatic women. HE4, a relatively new blood biomarker, has shown promise in the hospital setting. This study aimed to assess whether HE4 would improve ovarian cancer diagnosis in women with symptoms in primary care. We found combining HE4 levels with the currently used test (CA125) within an algorithm (Risk of Ovarian Malignancy Algorithm) improved the detection of ovarian cancer in primary care, particularly in women under 50 years of age, where diagnosis is more challenging. However, our results require validation in a larger sample. This study advances our knowledge of HE4 as an ovarian cancer biomarker in the primary care setting.

**Abstract:**

Human epididymis 4 (HE4) is a promising ovarian cancer biomarker, but it has not been evaluated in primary care. In this prospective observational study, we investigated the diagnostic accuracy of HE4 alone and in combination with CA125 for the detection of ovarian cancer in symptomatic women attending primary care. General practitioner (GP)-requested CA125 samples were tested for HE4 at a large teaching hospital in Manchester, and cancer outcomes were tracked for 12 months. We found a low incidence of ovarian cancer in primary care; thus, the cohort was enriched with pre-surgical samples from 81 ovarian cancer patients. The Risk of Ovarian Malignancy Algorithm (ROMA) was calculated using age (</>51) as a surrogate for menopause. Conventional diagnostic accuracy metrics were determined. A total of 1229 patients were included; 82 had ovarian cancer. Overall, ROMA performed best (AUC-0.96 (95%CI: 0.94–0.98, *p* = <0.001)). In women under 50 years, the combination of CA125 and HE4 (either marker positive) was superior (sensitivity: 100% (95%CI: 81.5–100.0), specificity: 80.1% (95%CI 76.7–83.1)). In women over 50, ROMA performed best (sensitivity: 84.4% (95%CI: 73.1–92.2), specificity: 87.2% (95%CI 84.1–90)). HE4 and ROMA may improve ovarian cancer detection in primary care, particularly for women under 50 years, in whom diagnosis is challenging. Validation in a larger primary care cohort is required.

## 1. Introduction

Ovarian cancer is the sixth most common cancer among women in the UK [1], with epithelial ovarian cancer accounting for 90% of cases. Ovarian cancer is the leading cause of mortality from gynaecological malignancy, resulting in an estimated 207,252 deaths worldwide in 2020 [2]. Overall 5-year survival rates are approximately 40% [3], and despite advances in ovarian cancer diagnosis and treatment over the last 40 years, survival rates have only improved by around 10%. Survival is dependent on the stage at diagnosis: women diagnosed with stage I disease have a 93% 5-year net survival rate compared to 13% in those diagnosed at stage IV [4]. However, early detection of ovarian cancer is challenging, with only 30% of cases currently diagnosed at stage I [5]. Screening studies have so far failed to demonstrate improved ovarian cancer survival outcomes, and, in the absence of effective screening strategies, the majority of women are diagnosed following presentation to primary care with relevant symptoms [6,7]. Detection of lower-volume disease increases rates of complete surgical resection, improving long-term survival [8].

The symptoms of ovarian cancer include bloating, abdominal pain and urinary frequency; these are non-specific and common in women who do not have cancer. Simple tests are needed to triage patients for urgent referral for specialist investigation or safe reassurance. In the United Kingdom (UK), it is recommended that general practitioners (GPs) perform a serum cancer antigen 125 (CA125) test in women with suspected ovarian cancer, particularly if they are over the age of 50 years [9]. However, CA125 is below the recommended threshold of 35 U/mL in 23% of women in primary care prior to ovarian cancer detection, which could contribute to delayed diagnosis [10]. In addition, CA125 can be raised in a number of benign conditions and other malignancies, with 90% of women in primary care with an abnormal CA125 found not to have ovarian cancer. Thus, symptomatic women with abnormal CA125 levels undergo unnecessary invasive investigations, with considerable cost and service implications for the healthcare system. Biomarkers that improve the diagnostic utility of CA125 could aid early detection and reduce unnecessary specialist investigations for ovarian cancer.

Human Epididymis 4 (HE4) is a whey acidic protein produced by the epithelium of the respiratory and reproductive tracts. HE4 is a promising biomarker for ovarian cancer, with approval in the USA from the US Food and Drug Administration (FDA) for remission monitoring [11]. Several studies have investigated HE4 as a diagnostic biomarker in secondary care, with reports indicating that HE4 has better specificity, and in some cases sensitivity, than CA125 [12,13,14,15,16]. In contrast to CA125, HE4 levels are less frequently affected by benign gynaecological conditions, in particular endometriosis [17]. This has led to increased interest in diagnostic models such as the Risk of Ovarian Malignancy Algorithm (ROMA), which calculates a woman’s risk of ovarian cancer using HE4, CA125 and menopausal status [12], with evidence suggesting a superior sensitivity compared to either CA125 or HE4 alone [13]. However, the evidence for HE4 in ovarian cancer detection is based on studies conducted in secondary care populations already known to have pelvic masses. The diagnostic performance of tests varies depending on the study population (the spectrum effect), and it is unclear whether these findings translate to a symptomatic primary care population, for whom new diagnostic approaches are urgently needed [18].

The aim of this study was to investigate the clinical utility of serum HE4 within a symptomatic primary care population. We hypothesised that HE4 would add to the diagnostic accuracy of CA125 in this setting.

## 2. Materials and Methods

### 2.1. Study Population

This study took the form of a prospective observational study in which women presenting to primary care who were tested for CA125 by their GP were also tested for HE4. The study was conducted at Manchester University NHS Foundation Trust (MFT). All GP-requested serum CA125 samples sent to the biochemistry laboratory at MFT between April 2018 and April 2019 were eligible for inclusion. Repeat samples and hospital-requested samples were excluded. Serum samples were tested for CA125 as requested, and then additionally tested for HE4. The CA125 result was available to the GP as normal.

Gynaecology clinics and multidisciplinary team meetings (MDT) were monitored in order to identify any women in the cohort referred to secondary care for further investigation. Women were approached at their clinic appointment and gave written informed consent to participate in the study. In addition, women with a raised HE4 but normal CA125 were invited to attend the gynaecology clinic for assessment and a pelvic ultrasound scan, to exclude ovarian cancer. Following assessment of 100 women with an isolated raised HE4 who were found not to have any significant pathology, this was discontinued (following appropriate ethical approval) to prevent unnecessary anxiety amongst women.

The clinical outcomes and final diagnoses of women in the cohort were identified from electronic hospital records at MFT and at the Christie NHS Foundation Trust Manchester. These hospitals manage all tertiary gynaecological oncology referrals in the region. This ensured that all diagnoses of ovarian cancer from our cohort would be identified. The primary outcome was the final diagnosis (all invasive epithelial ovarian, fallopian tube and primary peritoneal cancers) within 12 months of the GP-requested CA125. Where cancer was diagnosed, information on the tumour site, histological subtype, grade and stage were collected. Borderline ovarian tumours were excluded.

Demographic data included age and where the woman was referred to MFT, BMI, menopausal status, smoking history, hormone use, parity, family history, medical co-morbidities, medications and symptoms at presentation.

Due to the low incidence of ovarian cancer within our prospective primary care group, the sample set was enriched with pre-surgical serum samples from 82 women attending secondary care for primary surgical management of ovarian cancer. The diagnosis of ovarian cancer was made upon histological review of specimens by consultant pathologists who specialise in gynaecological oncology. Demographic data included age and BMI. Pathological data included FIGO 2014 stage, grade and histological sub-type. For clarity, we refer to women with a primary care-requested sample included in this study as the ‘primary care group’ and to the additional women with ovarian cancer who were tested pre-surgery as the ‘enrichment group’.

### 2.2. Laboratory Assays

GP-requested CA125 serum samples were identified by MFT laboratory staff. The samples underwent routine testing for CA125. CA125 was measured using an automated electrochemiluminescence Roche Cobas 6000 immunoassay analyser (601/602 modules). Testing was performed by MFT laboratory staff according to their standard operating procedure. HE4 levels are unaffected by the freeze-thaw process [19]; therefore, once tested for CA125, the samples were collected and stored in the laboratory freezer at −80 °C until HE4 analysis, enabling samples to be tested for HE4 in weekly batches. HE4 levels were measured using the Fujirebio Lumipulse G600II automated chemiluminescence enzyme assay (CLEIA) analyser in accordance with the manufacturer’s instructions. Samples from the enrichment cohort were stored at −80 °C until analysis and tested for CA125 and HE4 using the Fujirebio Lumipulse G600II.

The cut-off values for each biomarker were determined from the available literature and the manufacturers’ advice. The cut-off for CA125 was 35 U/mL [20]. The cut-off for HE4 in the study protocol was 70 pmol/L, as recommended in the literature [21]. However, this threshold is based on HE4 measurement using an enzyme immunoassay (EIA) method. The new CLEIA method has been shown to significantly overestimate levels of serum HE4 compared to EIA, and our previous work has identified equivalent cut-offs for the CLEIA method of 77 pmol/L [22]. These CLEIA cut-offs were used to improve comparability with published studies. Serum levels above these cut-offs were considered positive test results. Results were stratified and presented according to age (over and under 50 years).

**Figure 1:**
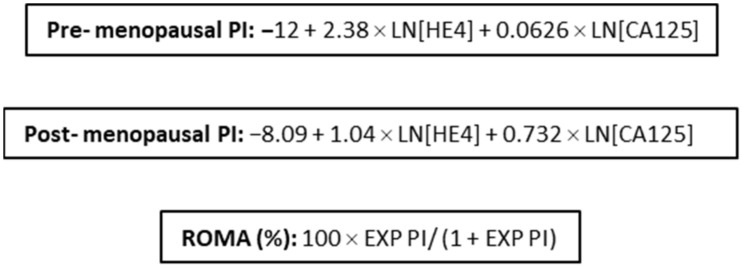
ROMA equations.

The ROMA score (Figure 1) was calculated for each sample. As menopausal status was unknown for the majority of study patients, age </≥ 51 years was used as a surrogate for menopausal status, as this is the average age of menopause in the UK [23]. Thresholds of 13.1% and 27.7% were used for women under and over the age of 51, respectively, as per the manufacturer [24].

### 2.3. Statistical Analysis

Based on limited published data regarding HE4 in a symptomatic primary care population [25], a power calculation estimated that a sample size of 1200 patients would have 90% power at the *p* = 0.05 level to detect differences in specificity for ovarian cancer detection between CA125 and HE4 of 5% or more, assuming >75% specificity.

Continuous data are reported as means with standard deviations and medians with interquartile ranges as appropriate. Comparison between two groups for continuous data was conducted using *t*-tests (Mann–Whitney U-tests used on data not normally distributed), and Kruskal–Wallis tests were used when comparing >2 groups. Chi-squared tests assessed the relationship between categorical data. To compare the overall diagnostic performance of CA125, HE4 and ROMA, receiver operator characteristic (ROC) curves were produced, and the area under the curve (AUC) was calculated for each test using the DeLong method with 95% confidence intervals (CI). Sensitivity and specificity were calculated by applying a CA125 cut-off of ≥35 U/mL and HE4 cut-offs of ≥77 pmol/L. For ROMA, cut-offs of 13.1% and 27.7% were used for women under and over the age of 51 years, respectively.

The correlation between age and serum HE4 levels was examined using Spearman’s rank correlation coefficients. Two quantile regression models predicting median HE4 values (to correct for the right-skewed distribution) were constructed using the serum results of those without a malignancy: Model 1 contained age as a continuous predictor, while Model 2 used age in 10-year bands (first and last bands wider to accommodate the smaller number at the tails of the distribution) treated as nominal variables, avoiding the assumption of a linear trend over these variables and allowing for the inspection of any threshold effects. The marginal distributions of these models were used to generate predicted median HE4 values with 95%CIs for different ages. We derived age-adjusted HE4 thresholds that maximised the product of sensitivity and specificity following the method of Liu [26] and using bootstrapping to obtain 95%CIs (1000 repetitions) separately for women aged under 50 years and those 50 years or over.

Data analyses were performed using STATA (StataCorp. 2015. Stata Statistical Software: Release 14. College Station, TX, USA: StataCorp LLC).

## 3. Results

### 3.1. Study Population

**Figure 2:**
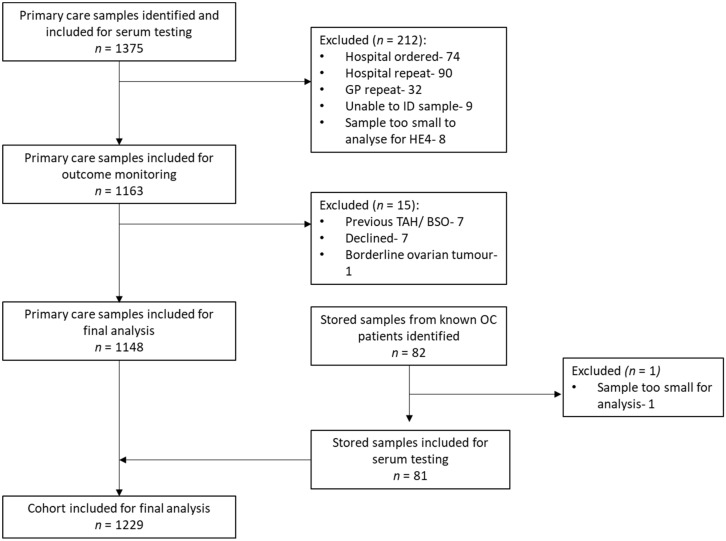
Study schema.

GP-requested serum samples from 1375 women were identified by the MFT laboratory, of which 1148 were eligible for inclusion in the final analysis (the primary care group). Of these, 1 was diagnosed with epithelial ovarian cancer. In total, 227 samples were excluded because they were hospital-ordered (*n* = 74), hospital repeats (*n* = 90), GP repeats (*n* = 32), too small a volume for testing (*n* = 8) or had no identifiable information (*n* = 9). A further 15 were excluded due to previous total hysterectomy and bilateral salpingo-oophorectomy (*n* = 7), declined participation (*n* = 7) or had a diagnosis of a borderline ovarian tumour on final histology (*n* = 1). In addition, stored serum samples from 82 epithelial ovarian cancer patients were identified to enrich the final cohort (the enrichment group). One of these patients was excluded as the serum sample was too small for testing, leaving 81 samples and bringing the total cohort to 1229, of whom 82 were women with ovarian cancer and 1147 were women without ovarian cancer (Figure 2).

The mean age of the whole group was 50 years (SD 15.7). Regardless of age, the women with ovarian cancer demonstrated significantly higher levels of CA125 (under 50 years: 14 vs. 167 U/mL (*p* < 0.001) and over 50 years: 11 vs. 257 U/mL (*p* < 0.001)) and HE4 (under 50 years: 53 vs. 284 pmol/L (*p* < 0.001) and over 50 years: 68 vs. 314 pmol/L (*p* < 0.001)), respectively (Table 1). In women without ovarian cancer, serum CA125 was significantly higher in those under 50 years (14 vs. 11 U/mL (*p* < 0.001)), whereas serum HE4 was significantly higher in women over 50 years (52 vs. 68 pmol/L (*p* < 0.001)). A total of 36% (190/525) of women without ovarian cancer over 50 years of age had an isolated raised serum HE4 compared to only 11% (65/622) of those under 50 years, suggesting age impacts serum HE4 levels. Serum HE4 was raised in isolation more frequently than serum CA125 regardless of age (22% (255/1147) vs. 5% (63/1147)).

#### 3.1.1. Clinico-Pathological Features of Malignancies

The clinico-pathological features of all epithelial ovarian cancers within the cohort are shown in Table 2. The mean age was 61 years (SD 12.3). Histological subtypes included: serous (57%), clear cell (10%), mucinous (10%), endometrioid (10%), mixed subtypes (5%) and carcinosarcoma (8%). Over two-thirds were high-grade (77%), advanced-stage (62%) disease at diagnosis. The ovarian cancer case diagnosed in the primary care group was an advanced-stage, high-grade serous tumour. Advancing grade and stage were associated with higher median CA125 (*p* = 0.04 and *p* < 0.001, respectively), whereas median HE4 was only found to be significantly higher in advancing stage (*p* < 0.001). HE4 was raised in isolation in 10 (12%) women with ovarian malignancy. The majority were high grade (90%) and early stage (70%) at diagnosis.

During the 12 months following the GP’s biomarker request, 15/1147 (1.3%) women in the group without ovarian cancer were diagnosed with a non-ovarian malignancy (Table S1). Site of malignancy included 2/15 (13%) breast, 4/15 (26%) colorectal, 3/15 (20%) endometrial, 3/15 (20%) lung, 1/15 (7%) renal, 1/15 (7%) cancer of unknown primary and 1/15 (7%) lymphoma. An isolated raised HE4 was observed in 6/15 (40%) malignancies, including lung (3/3), all of which were non-small cell lung cancers, renal (1/1), colorectal (1/4) and endometrial cancers (1/3). Four cancers (26%) had normal biomarkers; breast (2/2), endometrial (1/3) and colorectal cancers (1/4).

#### 3.1.2. Clinical Follow-Up

During the study, 101/1147 (9%) women with an isolated raised HE4 were invited to attend a gynaecology clinic to rule out malignancy (Table S2). The median age was 61.5 years (IQR 51-75). A high proportion were current (30/101, 29.7%) or ex-smokers (26/101, 25.7%). Hypertension was the most commonly reported co-morbidity (31/101, 31%); however, there was also a high number of women with chronic obstructive pulmonary disease (COPD) (14/101, 13.9%). The most common presenting complaint was abdominal/pelvic pain (61/101, 60%), followed by abdominal distension (23/101, 22.8%). A minority of women reported no symptoms indicative of ovarian cancer or gynaecological pathology (5/101, 5%). Following clinical examination and ultrasound imaging, no malignancies were diagnosed, and the majority of women had no demonstrable pathology (77/101, 76%). The most common benign gynaecological diagnosis was an ovarian cyst (10/101, 9.9%), of which 60% (6/10) were simple cysts, 10% haemorrhagic cysts (1/10) and 30% physiological cysts (3/10).

### 3.2. Test Diagnostic Accuracy

**Figure 3:**
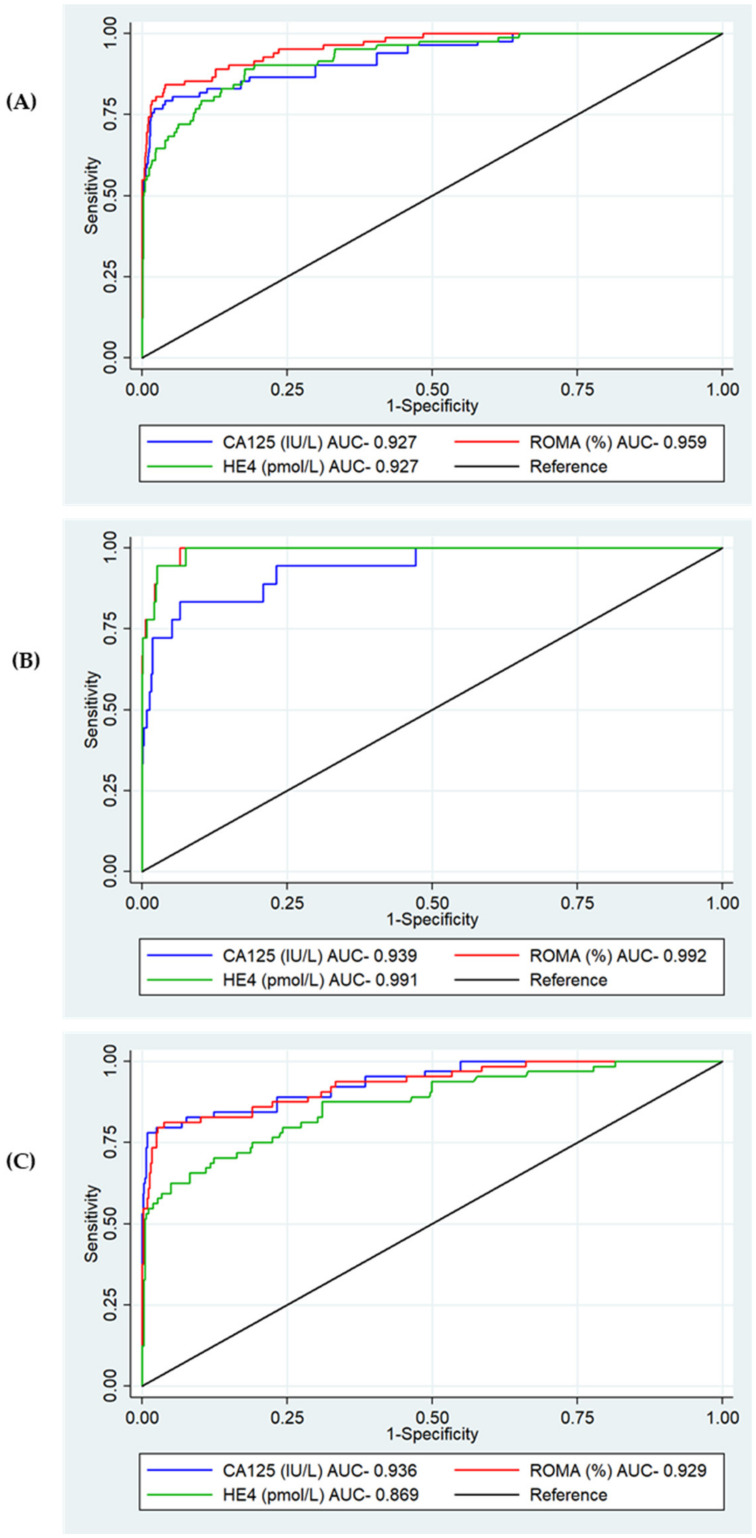
ROC analysis for serum CA125, HE4 and ROMA for the detection of epithelial ovarian cancer. (**A**) Overall. CA125 AUC 0.927 (95%CI 0.892–0.961), HE4 AUC 0.927 (95%CI 0.897–0.957), ROMA AUC 0.959 (95%CI 0.937–0.980) *p* = <0.001. (**B**) Under 50. CA125 AUC 0.939 (95%CI 0.881–0.996), HE4 AUC 0.991 (95%CI 0.982–1.000), *p* = 0.06. (**C**) Over 50. CA125 AUC 0.936 (95%CI 0.901–0.972), HE4 AUC 0.869 (95%CI 0.817–0.921), *p* < 0.001.

The total cohort (1229) was included in the biomarker diagnostic accuracy assessment for ovarian cancer. Overall, ROMA was observed to have the best diagnostic performance with an AUC of 0.959 (95%CI 0.94–0.98, *p* = <0.001) (Figure 3A). When stratified by age, HE4 demonstrated a better diagnostic performance than CA125 in women under 50 (AUC 0.991 vs. AUC 0.939, *p* = 0.06); however, in women over 50, CA125 was significantly better (AUC 0.936 vs. AUC 0.869, *p* < 0.0001) (Figure 3B,C).

The sensitivities and specificities of CA125, HE4 (alone and in combination) and ROMA for the detection of ovarian cancer are shown in Table 3. As a single biomarker, HE4 had a higher sensitivity than CA125 (90.2% (95%CI 81.7–95.7) vs. 80.5% (95%CI 70.3–88.4), respectively) but lower specificity (75.6% (95%CI 73.0–78.0) vs. 92.2% (95%CI 90.4–93.6), respectively). A combination of HE4 and CA125, using a strategy where either marker was positive, proved to have the best sensitivity (92.7%, 95%CI 84.8–97.3). However, this combination also demonstrated markedly reduced specificity (70.0%, 95%CI 67.3–72.6). Overall, ROMA provided the best balance between sensitivity and specificity (87.8% (95%CI 78.7–94.0) and 80.8% (95%CI 78.4–83.1), respectively). In our cohort, ROMA would lead to an additional 136 women being referred to secondary care compared to CA125, and of these, 6 (4%) would have an ovarian malignancy, suggesting that 1 in 23 of those who would be referred as a two-week wait would have ovarian cancer.

Due to the disparity in age between women with and without ovarian cancer and the impact that age has on serum HE4, sensitivity and specificity were also calculated stratified by age (under 50 years and over 50 years) (Table 4). In women under 50 years of age, the combination of HE4 and CA125 where either was positive performed the best, improving sensitivity (100%, 95%CI 81.5–100) but at a cost to specificity (80.1%, 95%CI 76.7–83.1), which, in our cohort would lead to an additional 67 referrals for further evaluation, of which 2 (3%) would have ovarian cancer (1 in 34), compared to using CA125 alone. The combination of CA125 and HE4 where either was positive also had the best sensitivity (90.6%, 95%CI 80.7–96.5) in women over the age of 50; however, this was at a significant cost to specificity (58.1%, 95%CI 53.7–62.4), which, compared to CA125, would lead to 197 extra referrals to secondary care, 1 in 28 of whom would have a malignancy (4%).

#### 3.2.1. Age and Adjusted Serum HE4 Thresholds

Adjusted median HE4 was predicted for different ages based on data from the primary care group and is shown in Table 5. The 15 women with non-ovarian malignancies and 2 with ovarian malignancies were excluded. The mean age was 49.4 years (SD 15.7).

Due to the impact of age on serum HE4 levels, age-adjusted cut-offs were estimated. Cut-offs of 68 pmol/L (95%CI: 50.45–86.44) and 96 pmol/L (95%CI: 68.19–124.11) maximised the product of sensitivity and specificity for women under and over 50 years of age, respectively. Table 6 displays the sensitivity and specificity of HE4 when using the estimated age-adjusted HE4 cut-offs applied to the total group (*n* = 1229). In women under the age of 50 years, the age-adjusted HE4 cut-off was found to have the same sensitivity as the previously used threshold of 77 pmol/L, but a lower specificity, both alone (80.4% (95%CI 77.0–83.4) vs. 88.4% (95%CI 85.6–90.8)) and in combination with CA125 (either positive: 73.5% (95%CI 69.8–76.9) vs. 80.1% (95%CI 76.7–83.1), both positive: 97.3% (95%CI 95.7–98.4) vs. 98.7% (95%CI 97.5–99.4)). Overall, in women over 50 years of age, the age-adjusted HE4 cut-off demonstrated a better balance of sensitivity and specificity compared to the threshold from the literature, with a superior specificity, both alone (74.7% (95%CI 70.7–78.3) vs. 60.4% (95%CI 56.1–64.6)) and in combination with CA125 (either positive: 71.8% (95%CI 67.8–75.6) vs. 58.1% (95%CI 53.7–62.4), both positive: 97.1% (95%CI 95.3–98.4) vs. 96.6% (95%CI 94.6–98.0)), and with a minimal reduction in sensitivity. Based on our data, a combination of CA125 and age-adjusted HE4 where either test was positive would lead to fewer additional referrals to secondary care than a threshold of 77 pmol/L (123 vs. 197) and would diagnose an additional 5 malignancies (1 in 25) compared to CA125; this would be 2 fewer than a threshold of 77 pmol/L.

#### 3.2.2. Test Diagnostic Accuracy by Stage at Diagnosis

**Figure 4:**
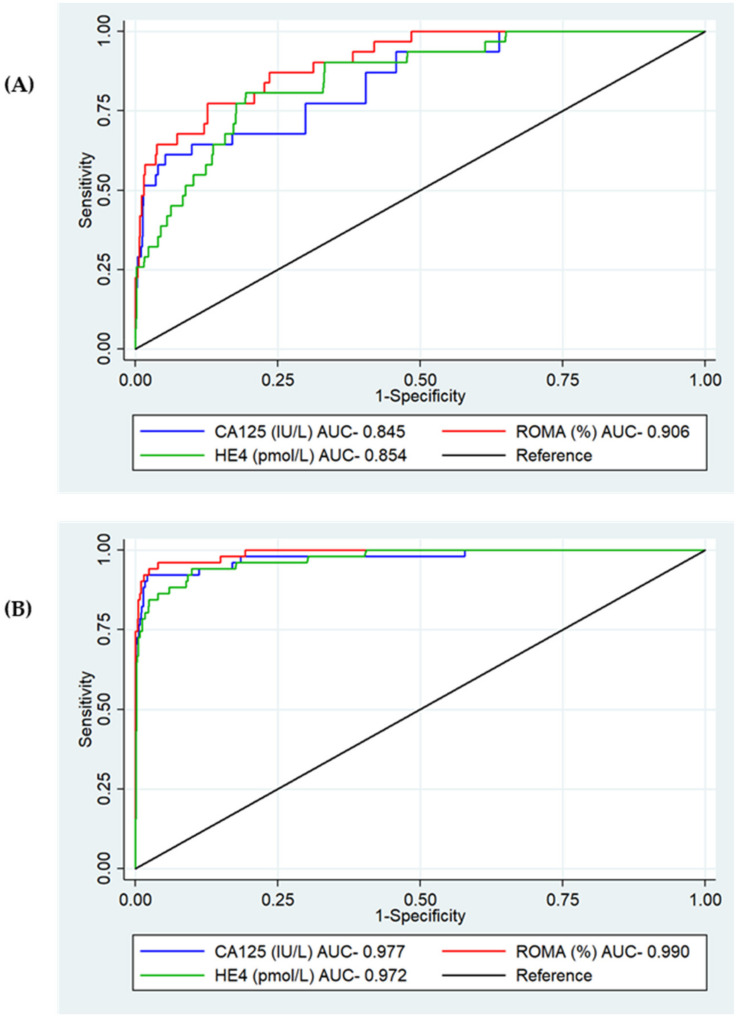
ROC analysis for serum CA125, HE4 and ROMA for the detection of epithelial ovarian cancer by stage at diagnosis. (**A**) Early stage. CA125 AUC 0.845 (95%CI 0.771–0.919), HE4 AUC 0.854 (95%CI 0.791–0.917), ROMA AUC 0.906 (95%CI 0.856–0.956), *p* < 0.001. (**B**) Late stage. CA125 AUC 0.977 (95%CI 0.953–1.000), HE4 AUC 0.972 (95%CI 0.951–0.993), ROMA AUC 0.990 (95%CI 0.981–1.000), *p* = 0.006.

Of the 82 ovarian malignancies, 31 (38%) were diagnosed at an early stage (stage I+II), and 51 (62%) were diagnosed at an advanced stage (stage III + IV). ROMA had the best diagnostic performance for both early- and late-stage disease, with an AUC of 0.906 (95%CI 0.856–0.956, *p* < 0.001) and 0.990 (95%CI 0.981–0.999, *p* = 0.006), respectively (Figure 4A,B). There was no difference in the performance of CA125 and HE4 for either early- or late-stage detection.

The sensitivity and specificity of HE4, CA125 and ROMA for diagnosing early- and late-stage disease are shown in Table 7. At the thresholds examined, HE4 demonstrated a better sensitivity (80.6% (95%CI 62.5–92.5)) for diagnosing early-stage disease compared to CA125 (61.3% (95%CI 42.2–78.2)). When used together, the combination of CA125 and HE4 where either was positive improved the sensitivity to 83.9% (95%CI 66.3–94.5) and had a superior sensitivity compared to ROMA (74.3% (55.4–88.1)). There was, however, a marked reduction in specificity compared to CA125 alone (70.0% (67.3–72.6) vs. 92.2% (95%CI 90.4–93.6)). CA125 alone had the best overall balance of sensitivity and specificity for the detection of advanced disease, and whilst ROMA increased the sensitivity to 96.1% (95%CI 86.5–99.5), this was at a cost to the specificity (80.8% (95%CI 78.4–83.1)).

## 4. Discussion

### 4.1. Summary

In this study, we examined the diagnostic performance of HE4 alone and in combination with CA125 for the detection of ovarian cancer in a cancer-enriched symptomatic primary care population. We found that the combination of markers using ROMA performed the best overall. When stratified by age, HE4 performed better in women under 50, whereas CA125 was superior in women over 50. The addition of HE4 to CA125 using a strategy where either was positive improved sensitivity compared to CA125 alone; however, this was at a cost to specificity. In women under 50, this combination performed well overall, with age-adjusted thresholds adding little benefit. However, in women over the age of 50, the improved sensitivity of combining the markers came at a significant cost to specificity. Age-adjusted thresholds improved the specificity and provided improved overall accuracy; however, ROMA appeared to perform the best in the older population. At the thresholds examined, HE4 had a better sensitivity for the detection of early-stage ovarian cancer compared to CA125, and the combination of the two further improved the sensitivity, but at a cost to specificity. Overall, these data suggest that HE4 in combination with CA125 may improve ovarian cancer detection in primary care, particularly in younger women, in whom diagnosis can be challenging. Furthermore, ROMA may improve detection in older women; however, confidence intervals were wide, and our case numbers were small. Therefore, a larger primary care study is needed to further evaluate the markers within the primary care setting.

### 4.2. Strengths and Limitations

To our knowledge, this is the first study investigating the utility of HE4 and ROMA in primary care. A major strength of this study is that our control group was primary care women who had been selected by their GP for ovarian cancer tests, i.e., the population of interest. Although studies have previously evaluated HE4 in secondary care populations and in case-control studies using healthy controls, the performance of tests (including the sensitivity and specificity) is affected by the population in which it is used (the spectrum effect) [18]. Women selected for ovarian cancer testing in primary care who ultimately do not have the disease have a variety of other pathologies which can affect biomarker levels and which will differ in severity and prevalence from women in secondary care studies. Therefore, the inclusion of over 1000 women selected for ovarian cancer testing by their GP should provide a more accurate assessment of HE4, CA125 and ROMA specificity within the real-world primary care setting. Another strength of this study is that we were able to clinically assess 100 women with isolated elevations in HE4, exclude malignancy and identify likely causes for biomarker elevation where present.

A limitation of this study is that there was a lower incidence of ovarian cancer within the primary care cohort than anticipated based on previous studies, necessitating the inclusion of additional stored samples from women with ovarian cancer in order to adequately assess marker sensitivity. It is possible that, as these serum samples were collected in secondary care prior to surgery rather than in primary care, the disease profile may be more advanced than in primary care. However, it is reassuring that the stage distribution and the sensitivity of CA125 in this study are similar to that of a recently published large primary care cohort study on CA125 diagnostic accuracy [10]. It is likely that the sensitivities reported will be impacted by the secondary care enrichment group, and in reality, we would expect them to be slightly lower if all the ovarian cancer cases were diagnosed from primary care. Nonetheless, our study is likely to provide more accurate information on how HE4 would perform in primary care than studies conducted solely in secondary care populations, suggesting further evaluation would be of value within this setting. However, to prospectively evaluate the sensitivity of HE4 and ROMA in primary care, a much larger population sample with a larger case mix of epithelial ovarian cancer histological subtypes would be required.

We were able to collect limited clinical and demographic data for many of the women in our cohort, as the majority had normal biomarkers and did not present to secondary care. Therefore, while the primary care cohort was selected for ovarian cancer testing by GPs due to clinical suspicion, the symptoms or signs that triggered testing could not be determined in this study. Furthermore, for the large majority of women, we were unable to determine menopausal status, which meant we had to use age as a substitute when calculating ROMA. This will impact the accuracy of the results reported for ROMA, and is a major limitation. However, the promising results suggest further evaluation is warranted, as the correct menopausal status will likely increase accuracy. Furthermore, whilst we have identified all patients in the primary care cohort diagnosed with ovarian cancer within Greater Manchester in the 12 months post-testing, it is possible that women may have moved and been diagnosed in another region or have taken longer than 12 months to be diagnosed.

### 4.3. Comparison with Existing Literature

Serum HE4 has shown promise for clinical use in secondary care, both for ovarian cancer diagnosis and recurrence monitoring [11]. A principal reported advantage of HE4 over CA125 is that levels are not affected by endometriosis, which could contribute to higher test specificity, in particular amongst younger women [17]. This has led to the development of several algorithms incorporating HE4 to classify women at high or low risk of ovarian cancer; most notably ROMA, which combines CA125, HE4 and menopausal status [12]. The diagnostic performance of ROMA in our study is comparable to that in the literature and was found to be superior to either marker alone. Two meta-analyses suggested a superior performance of ROMA compared to HE4 and CA125 alone. Li et al. reported an AUC of 0.93 (95%CI 0.90–0.95) and a sensitivity and specificity of 89% and 83%, respectively for ROMA in a population of 7792 women [27]. Similarly, Dayyani et al. reported a sensitivity of 87% and a specificity of 85% for ROMA, with an overall AUC of 0.921 (95%CI 0.855–0.960) in a population of 1975 women [13]. However, there is heterogeneity amongst studies, with few directly comparing performance to the currently used Risk of Malignancy Index (RMI). This has led the National Institute of Clinical Excellence (NICE) to conclude that there was not enough evidence to support the diagnostic accuracy of ROMA and its routine use in clinical care [24]. Two UK-based studies are ongoing that attempt to establish and compare the accuracy of current and new biomarkers, ultrasound scores and risk prediction models [28,29]. There are significant differences between our population and those in the literature, with all the studies being conducted in secondary care in women who have had ultrasound imaging and a known pelvic mass. No models incorporating HE4 have been evaluated in primary care; however, our data suggest that larger prospective primary care studies evaluating ROMA would be of value, given its superior performance.

The addition of HE4 to CA125 in our study, both in combination (<50 years) and within the context of ROMA, improved the diagnostic accuracy of CA125 alone in a primary care population. Unexpectedly, we observed that HE4 had a lower specificity (75.6%) in primary care compared to that reported in secondary care studies (87%) [30]. This may be due to differences in population between our study (which included primary care patients) and existing studies, as test accuracy varies with the incidence and characteristics of a population [18]. The population of symptomatic women presenting to primary care is diverse, with wide variation in age, physiological characteristics, lifestyle characteristics and co-morbidities, all of which may impact HE4 levels. In women without ovarian cancer, we found that 22% (255/1147) had an isolated HE4 above the threshold, which was much higher than for CA125 (5%, 63/1147). This may be attributed to the impact of physiological confounders on circulating levels of HE4 in the general population. Due to the potential benefit of HE4 in primary care, we reviewed 100 women with an isolated raised HE4 to rule out ovarian cancer. The majority of women were found to have no pathology on clinical assessment or scan (76%), and whilst there were several different benign pathologies identified, the most common of which was a benign ovarian cyst (9.9%), none required intervention. No malignancies were diagnosed. Whilst HE4 is overexpressed in several benign ovarian cyst tissues, the serum levels of HE4 are rarely elevated [31]. Moore et al. found that serum HE4 was raised in only 7% of those with benign ovarian cysts, compared to CA125, which was raised in 29% [32]. Over half of our population with isolated raised HE4 were either current smokers or ex-smokers, much higher than the UK prevalence reported for those of the same age (median age 61 years), which ranges from 7.9–14.5% [33]. Smoking is known to affect serum concentrations of HE4, with levels around 20–30% higher in smokers compared to non-smokers [34]. In addition, we found a high proportion had a history of COPD (13.9%), a prevalence significantly higher than that reported in the UK for ages 61–77 (5.3%) [35]. HE4 was found to be raised in 73% (11/15) of all malignancies diagnosed in the primary care group and was raised in isolation in 47%, particularly in the non-small cell lung cancers and transitional cell renal cancer, which is similar to previous reports [31]. HE4 is expressed in respiratory epithelia as well as reproductive epithelia and therefore has been investigated as a biomarker for lung cancer, with a sensitivity of 65% and specificity of 88% [36]. HE4 was raised in two of the three endometrial cancers diagnosed in our cohort and was raised in isolation in one of these. This is in keeping with evidence suggesting HE4 might be a promising diagnostic and prognostic marker in endometrial cancer [37]. Interestingly, HE4 was also elevated in 50% of colorectal cancers. Few studies have evaluated HE4 as a biomarker in colorectal cancer; however, a study by Kemal et al. suggested it may be of use in patients with stage III and IV disease [38]. These observations emphasise the importance of considering other malignancies when HE4 is raised but ovarian cancer has been ruled out.

We demonstrated that HE4 has a much lower specificity in an older population and that levels increase with advancing age, an association that has also been found in other non-cancer populations [39,40,41], suggesting HE4 may be less useful in older women. Bolstad et al. reported average serum HE4 levels in a healthy population were increased by 2% at 30 years, 9% at 40 years, 20% at 50 years, 37% at 60 years, 63% at 70 years and 101% at 83 years compared to those aged 20 years [39]. This led us to develop age-adjusted thresholds for HE4 to investigate whether this might improve the diagnostic accuracy in women under and over the age of 50 years. In women under 50 years, age-adjusted cut-offs added little benefit to cut-offs derived from the literature; however, in women over 50 years, age-adjusted thresholds improved the specificity of HE4 both alone and in combination, suggesting that there may be a role for age adjustment in women over 50 years. In our cohort, ROMA performed better than age-adjusted thresholds in this group and, in our study, is already adjusted for age (51 years). It would be interesting to evaluate the performance of ROMA when calculated using menopausal status compared to an age-adjusted HE4 and CA125 combination and would be worth exploring in a much larger cohort to investigate if there is any true significant clinical benefit over CA125 alone.

### 4.4. Clinical and Research Implications

CA125 is recommended as a first-line test for ovarian cancer in women presenting with possible symptoms of the disease in a number of countries [42], with an ultrasound advocated if CA125 is abnormal. Specialist referral is then made only if the ultrasound is abnormal in addition to CA125 [9]; however, there are a number of women with false-negative CA125, with evidence suggesting that diagnosis in this group took twice as long compared to those with a raised CA125 [43]. Delays in diagnosis impact early detection and survival. Indeed, improvement in the numbers of cancers detected early in primary care through increased access to tests was found to be a priority for patients [44]. A recent study showed that CA125 has reasonable sensitivity (85%) and specificity (94%) for invasive ovarian cancer when used in primary care [10,43]; however, our study suggests the addition of HE4 would further improve sensitivity, with ROMA detecting 1 ovarian cancer case for every additional 23 women referred for further assessment. Furthermore, ROMA had significantly better sensitivity for the detection of early-stage disease compared to CA125 alone.

Ovarian cancer is most commonly diagnosed in post-menopausal women; however, an estimated 3–17% of cases are diagnosed in women under the age of 40, and 40% of primary care CA125 tests are requested in women under 50 [45,46]. In the UK, the incidence of ovarian cancer in women of reproductive age ranges from 2.1–19.4 per 100,000, doubling to 41.6 per 100,000 in women aged between 60–64 years [47], and the incidence in primary care is further reduced. Only a small proportion of these are epithelial ovarian cancers, with malignant germ cell, sex cord-stromal tumours and borderline ovarian tumours a more common diagnosis. The low incidence of epithelial ovarian cancer in this population, vague symptoms and the influence of common benign gynaecological conditions on CA125 make early detection challenging. We have shown that HE4 has a better diagnostic performance than CA125 in younger women, and the combination of HE4 and CA125 detected all ovarian cancers (100% sensitivity) in women under 50 years, leading to the detection of 1 extra case for every additional 34 women referred to secondary care.

Whilst our data suggests that the addition of HE4 to CA125, either as a combination where either is positive or in the context of ROMA, would be of benefit to current primary care diagnostic pathways, a large primary care-based study would be needed to externally validate these cut-offs and assess the potential clinical and health economic implications of implementing them within primary care.

This study has wider implications for biomarker research. We evaluated a promising biomarker that had performed well in other settings. When translated to a primary care population, it exhibited different performance metrics. This highlights the need to evaluate novel biomarkers within the intended population prior to their clinical use within that setting. This has not always been the case: the accuracy of CA125 was only recently evaluated in primary care, and there is limited evidence on the accuracy of prostate-specific antigen (PSA) in symptomatic men in primary care despite its widespread use in this context [48]. This study also demonstrates the challenges of evaluating diagnostic biomarkers in low-prevalence populations, given the large numbers of patients that need to be recruited in order to adequately assess test sensitivity.

## 5. Conclusions

Secondary care ovarian cancer studies have reported that HE4 is a promising diagnostic marker for ovarian cancer, and our findings suggest it may also be of benefit in addition to CA125 for the detection of ovarian cancer in a primary care population. ROMA improved diagnostic accuracy overall, in women over the age of 50 years, and for detection of early-stage disease. Furthermore, our data suggest HE4 may be of particular benefit in the detection of women under the age of 50 and in whom diagnosis is challenging due to a low incidence and poor diagnostic accuracy of CA125 alone. A larger study would be needed to validate our findings and to examine the potential benefits and cost implications of the additional test.

## Figures and Tables

**Table 1:** Summary of biomarker and eGFR levels in women with and without ovarian cancer, stratified by age.

Variable	No Ovarian Cancer		Ovarian Cancer	
	*N* = 1147		*N* = 82	
	Age < 50 Years	Age ≥ 50 Years	Age < 50 Years	Age ≥ 50 Years
	*N* = 622	*N* = 525	*N* = 18	*N* = 64
**Age (years)**				
Mean (SD)	37 (8.6)	63 (10.1)	44 (4.0)	65 (9.5)
**Serum CA125 (U/mL)**				
Median (IQR)	14 (10–21)	11 (8–17)	167 (50–2352)	257 (85–961)
Negative result (*n*, %)	562 (90)	495 (94)	3 (17)	13 (20)
Positive result (*n*, %)	60 (10)	30 (6)	15 (83)	51 (80)
**Serum HE4 (pmol/L)**				
Median (IQR)	53 (45–65)	68 (55–96)	284 (150–534)	314 (107–742)
Negative result (*n*, %)	550 (88)	317 (60)	0 (0)	8 (12)
Positive result (*n*, %)	72 (12)	208 (40)	18 (100)	56 (88)
**Combined results (*n*, %)**				
Negative CA125 + HE4	498 (80)	305 (59)	0 (0)	6 (9)
Positive CA125, negative HE4	51 (8)	12 (2)	0 (0)	2 (3)
Positive HE4, negative CA125	65 (11)	190 (36)	3 (17)	7 (11)
Positive CA125 + HE4	8 (1)	18 (3)	15 (83)	49 (77)

SD—standard deviation. *n*—number. IQR—interquartile range.

**Table 2:** Clinico-pathological features and serum results of ovarian cancer cases (*n* = 82).

Variable	Number (%)	Median CA125 (IQR), U/mL	Median HE4 (IQR), pmol/L
**Histological Classification**			
Serous	47 (57)	708 (97–2352)	411 (186–830)
Clear cell	8 (10)	206 (21–5488)	135 (88–344)
Mucinous	8 (10)	100 (40–210)	90 (74–134)
Endometrioid	8 (10)	169 (22–468)	404 (88–4049)
Mixed	4 (5)	132 (62–216)	311 (124–441)
Carcinosarcoma	7 (8)	279 (80–978)	280 (112–804)
**Tumour Grade**			
1	15 (18)	105 (24–219)	139 (69–207)
2	4 (5)	374 (69–2209)	422 (100–25617)
3	63 (77)	526 (92–2315)	377 (137–804)
**FIGO 2014 Stage**			
1	25 (30)	90 (17–185)	117 (89–197)
2	6 (8)	193 (13–3435)	257 (58–2695)
3	33 (40)	746 (162–2546)	418 (186–828)
4	18 (22)	806 (174–2948)	518 (281–981)

SD—standard deviation. IQR—interquartile range. *n*—number.

**Table 3:** Overall sensitivity and specificity of serum HE4, CA125 (alone and in combination) and ROMA for the detection of ovarian cancer.

*N* = 1229 OC = 82 (7%)	Sensitivity % (95%CI)	Specificity % (95%CI)	True Positive	False Positive
CA125 (≥35 U/mL)	80.5 (70.3–88.4)	92.2 (90.4–93.6)	66	90
HE4 (≥77pmol/L)	90.2 (81.7–95.7)	(73.0–78.0)	74	280
Combination, either positive	92.7 (84.8–97.3)	(67.3–72.6)	76	344
Combination, both positive	78.0 (67.5–86.4)	97.7 (96.7–98.5)	64	26
ROMA	87.8 (78.7–94)	80.8 (78.4–83.1)	72	220

CI—confidence interval. *n*—number. OC—ovarian cancer. PPV—positive predictive value.

**Table 4:** Sensitivity and specificity of serum HE4, CA125 (alone and in combination) and ROMA for the detection of ovarian cancer stratified by age under and over 50 years.

	Sensitivity % (95%CI)	Specificity % (95%CI)	True Positive	False Positive
a. Under 50 years of age (*n* = 640, OC = 18)				
CA125 (≥35U/mL)	83.3 (58.6–96.4)	90.4 (87.8–92.6)	15	60
HE4 (≥77pmol/L)	100.0 (81.5–100.0)	88.4 (85.6–90.8)	18	72
Combined, either positive	100.0 (81.5–100.0)	80.1 (76.7–83.1)	18	124
Combined, both positive	83.3 (58.6–96.4)	98.7 (97.5–99.4)	15	8
ROMA	100.0 (81.5–100.0)	75.4 (71.8–78.7)	18	153
b. Over 50 years of age (*n* = 589, OC = 64)				
CA125 (≥35U/mL)	79.7 (67.8–88.7)	94.3 (91.9–96.1)	51	30
HE4 (≥77pmol/L)	87.5 (76.8–94.4)	60.4 (56.1–64.6)	56	208
Combined, either positive	90.6 (80.7–96.5)	58.1 (53.7–62.4)	58	220
Combined, both positive	76.7 (64.3–86.2)	96.6 (94.6–98.0)	49	18
ROMA	84.4 (73.1–92.2)	87.2 (84.1–90)	54	67

CI—confidence interval. *n*—number. OC—ovarian cancer. PPV—positive predictive value.

**Table 5:** Impact of age on serum HE4 using quantile regression.

	Predicted Median HE4 (95%CI) pmol/L
Age at the following years	
20	47.2 (43.6–50.8)
30	52.1 (49.5–54.8)
40	57.0 (55.2–58.9)
50	62.0 (60.5–63.5)
60	66.9 (65.0–68.8)
70	71.8 (69.1–74.6)
80	76.8 (73.0–80.5)
Age in bands (years)	
<30	53.4 (48.8–58.0)
30–39	55.4 (51.4–59.5)
40–49	55.4 (52.4–58.5)
50–59	60.8 (57.6–64.0)
60–69	69.5 (65.0–74.1)
≥70	88.5 (83.5–93.5)

CI—confidence interval. NA—not applicable.

**Table 6:** Sensitivity and specificity of HE4 using age-adjusted cut-offs for the diagnosis of ovarian cancer in women over and under 50 years of age.

	Sensitivity % (95%CI)	Specificity % (95%CI)	True Positive	False Positive
a. Under 50 years of age (*n* = 640, OC = 18)				
CA125 (≥35 U/mL)	83.3 (58.6–96.4)	90.4 (87.8–92.6)	15	60
HE4 (≥68 pmol/L)	100 (81.5–100)	80.4 (77.0–83.4)	18	122
Combined, either positive	100 (81.5–100)	73.5 (69.8–76.9)	18	165
Combined, both positive	83.3 (58.6–96.4)	97.3 (95.7–98.4)	15	17
b. Over 50 years of age (*n* = 589, OC= 64)				
CA125 (≥35 U/mL)	79.7 (67.8–88.7)	94.3 (91.9–96.1)	51	30
HE4 (≥96 pmol/L)	79.7 (67.8–88.7)	74.7 (70.7–78.3)	51	133
Combined, either positive	87.5 (76.8–94.4)	71.8 (67.8–75.6)	56	148
Combined, both positive	71.9 (59.2–82.4)	97.1 (95.3–98.4)	46	15

CI—confidence interval. PPV—positive predictive value.

**Table 7:** Sensitivity and specificity of serum HE4, CA125 (alone and in combination) and ROMA for the detection of early- and late-stage ovarian cancer.

	Early Stage (I + II) *n* = 31		Late Stage (III + IV) *n* = 51	
	Sensitivity % (95%CI)	Specificity % (95%CI)	Sensitivity % (95%CI)	Specificity % (95%CI)
CA125 (≥35 U/mL)	61.3 (42.2–78.2)	92.2 (90.4–93.6)	92.2 (81.1–97.8)	92.2 (90.4- 93.6)
HE4 (≥77 pmol/L)	80.6 (62.5–92.5)	75.6 (73.0–78.0)	96.1 (86.5–99.5)	75.6 (73.0–78.0)
Combined, either positive	83.9 (66.3–94.5)	70.0 (67.3–72.6)	98.0 (89.6–100.0)	70.0 (67.3–72.6)
Combined, both positive	58.1 (39.1–75.5)	97.7 (96.7–98.5)	90.2 (78.6–96.7)	97.7 (96.7–98.5)
ROMA	74.3 (55.4–88.1)	80.8 (78.4–83.1)	96.1 (86.5–99.5)	80.8 (78.4–83.1)

CI—confidence interval. *n*—number

## Data Availability

Fully anonymised data are available on reasonable request to the corresponding author.

## Supplementary Materials

The following supporting information can be downloaded at: https://www.mdpi.com/article/10.3390/cancers14092124/s1, Table S1: Malignancies diagnosed within 12 months of index CA125 test in primary care group, Table S2: Clinico-demographics of the cohort referred to hospital for investigation with a raised serum HE4 (*n* = 101).
