# Supplementary material for: The Performance of HE4 Alone and in Combination with CA125 for the Detection of Ovarian Cancer in an Enriched Primary Care Population
Source: Cancers (Basel). 2022 Apr 24;14(9):2124. doi: 10.3390/cancers14092124 (PMC9101616; doi:10.3390/cancers14092124)
Supplement: Supplementary file 1 [file cancers-14-02124-s001.zip › cancers-1663641-supplementary.pdf]

cancers

MDPI

*Supplementary Materials*

# The Performance of HE4 Alone and in Combination with CA125 for the Detection of Ovarian Cancer in an Enriched Primary Care Population

**Chloe E. Barr, Garth Funston, David Jeevan, Sudha Sundar, Luke T. A. Mounce and Emma J. Crosbie**

**Table S1.** Malignancies diagnosed within 12 months of index CA125 test in primary care group.

| ID no ($n$ = 17) | Tumour Site | Histological subtype | Grade | Stage | CA125 ≥ 35 U/mL | HE4 ≥ 77 pmol/L |
|---|---|---|---|---|---|---|
| 3 | Breast | Invasive ductal | 2 | T2, N0, M0 | No | No |
| 254 | Breast | Mixed invasive lobular and ductal | 2 | Lobular- T2, N0, M0<br>Ductal- T1, N0, M0 | No | No |
| 459 | Colorectal | Adenocarcinoma | 2 | T4, N0, M0 | No | No |
| 29 | Colorectal | Adenocarcinoma | 2 | T3, N0, M0 | Yes | Yes |
| 996 | Colorectal | Adenocarcinoma | 3 | T4a, N2b, M0 | Yes | No |
| 1130 | Colorectal | Adenocarcinoma | 2 | T3, N0, M0 | No | Yes |
| 455 | Lung | Non-small cell | NK | T1C, N0, MO | No | Yes |
| 696 | Lung | Non-small cell | NK | T1A, N0, M0 | No | Yes |
| 1010 | Lung | Non-small cell | NK | T1c, N0, M0 | No | Yes |
| 64 | Endometrial | Endometrioid | 1 | 1A | No | No |
| 580 | Endometrial | Endometrioid | 3 | 1B | Yes | Yes |
| 1214 | Endometrial | Endometrioid | 1 | 1A | No | Yes |
| 436 | Ovarian | Borderline serous | NA | 1C | Yes | No |
| 542 | Ovarian | Serous | 3 | 4B | Yes | Yes |
| 967 | Renal | Transitional cell carcinoma | NK | T4b, N2, M0 | No | Yes |
| 1081 | Unknown Primary | Adenocarcinoma | 3 | 4B | Yes | Yes |
| 1221 | Lymphoma | Extra nodal marginal zone | NK | NK | No | Yes |

n- number. NK- not known

**Table S2.** Clinico-demographics of the cohort referred to hospital for investigation with a raised serum HE4 ($n$ = 101).

| Clinico- demographic variable | Number (%) |
|---|---|
| **Age (years)** | |
| Median (IQR) | 61.5 (51–75) |
| **BMI (kg/m²)** | |
| Median (IQR) | 26 (23–31) |
| **Ethnicity** | |
| White | 74 (73.3) |
| Black | 6 (5.9) |
| Asian | 18 (17.8) |
| Mixed | 0 (0) |
| Other | 3 (3) |
| **Smoking status** | |
| Non- smoker | 45 (44.6) |
| Ex- smoker | 26 (25.7) |
| Current smoker | 30 (29.7) |
| **Menopausal status** | |
| Pre- menopausal | 22 (21.8) |
| Post- menopausal | 79 (78.2) |
| **Parity** | |
| Nulliparous | 18 (17.8) |
| Multiparous | 83 (82.2) |
| **Co-morbidities** | |

| | |
|---|---|
| **Cardiac** | |
| Hypertension | 31 (30.7) |
| Atrial Fibrillation | 2 (2) |
| Heart Failure | 3 (3) |
| Ischaemic heart disease | 8 (7.9) |
| **Respiratory** | |
| Asthma | 10 (9.9) |
| COPD | 14 (13.9) |
| **Endocrine** | |
| Diabetes Mellitus | 12 (11.9) |
| Hypothyroidism | 12 (11.9) |
| **Renal** | |
| Chronic kidney disease | 1 (1) |
| **Gynaecological** | |
| Endometriosis | 3 (3) |
| Fibroids | 1 (1) |
| PCOS | 2 (2) |
| **Other Previous Malignancy** | |
| Breast | 6 (5.9) |
| Lymphoma | 1 (1) |
| Renal | 1 (1) |
| **Presenting Complaint** | |
| **Ovarian Cancer symptom**[1] | |
| Abdominal/ pelvic pain | 61 (60.4) |
| Abdominal distension | 23 (22.8) |
| Satiety/ loss appetite | 3 (3) |
| Increased urinary urgency/ frequency | 4 (4) |
| **Other symptoms related to ovarian cancer**[1] | |
| Unexplained weight loss | 13 (12.9) |
| Fatigue | 3 (3) |
| Change in bowel habit | 5 (5) |
| **General Gynaecological symptom** | |
| Post-menopausal bleeding | 3 (3) |
| Uro-genital prolapse | 1 (1) |
| Vaginal discharge | 1 (1) |
| Intermenstrual bleeding | 3 (3) |
| Secondary amenorrhoea | 2 (2) |
| Dysmenorrhoea | 2 (2) |
| **Other** | |
| Hair loss | 1 (1) |
| Abnormal Blood Glucose | 1 (1) |
| Anaemia | 1 (1) |
| Foot swelling | 1 (1) |
| Rash | 1 (1) |
| **Final Diagnosis** | |
| **No Pathology** | 77 (76.2) |
| **Benign Gynae pathology** | |
| Ovarian cyst | 10 (9.9) |
| Endometrial polyp | 3 (3) |
| Atrophy | 1 (1) |
| Hydrosalpinx | 1 (1) |
| Fibroids | 2 (2) |
| Endometriosis | 1 (1) |
| Uro-Genital prolapse | 1 (1) |
| Dysfunctional uterine bleeding | 1 (1) |
| **Other** | |
| Adnexal fibrosis | 1 (1) |
| Constipation | 1 (1) |
| Chronic urinary retention | 1 (1) |
| Did not attend investigation | 1 (1) |
| **Malignancy** | 0 (0) |

[1] As per the NICE Guidelines (4). IQR- interquartile range. n- number
